# Editorial: Special 2021 Frontiers in Endocrinology collection for the 100^th^ anniversary of insulin discovery

**DOI:** 10.3389/fendo.2023.1181524

**Published:** 2023-03-20

**Authors:** Pierre De Meyts, Jeff M. P. Holly

**Affiliations:** ^1^ Cell Signalling Pole, de Duve Institute, Université Catholique de Louvain, Brussels, Belgium; ^2^ Bristol Medical School, University of Bristol, Southmead Hospital, Bristol, United Kingdom

**Keywords:** editorial, insulin, 100th anniversary, diabetes, obesity, pituitary adenomas, thyroid

To commemorate the 100th anniversary of the discovery and successful purification of insulin (1) at the University of Toronto by Banting, Best, Collip and McLeod in 1921 (earning the Nobel Prize to Banting and McLeod in 1923), Frontiers in Endocrinology organized a special collection related to insulin and insulin-like peptides, with Jeff M.P. Holly and Pierre De Meyts as supervisory editors. The aim was to bring together a collection of articles to celebrate the anniversary and the continued breadth of studies of this remarkable hormone. The articles could be submitted to the various sections of the journal.

The review by Irwin discusses the evolution of the insulin gene in the superfamily of insulin-like genes in multicellular invertebrates and vertebrates, as well as the recent discovery of insulin-like peptides in some fish viruses (2). Initial efforts to study the evolutionary aspects of insulin-like sequences relied on the sequencing of isolated peptides, but in the past 20 years, an increasing number of protein sequences have been predicted from the complete genome sequences of organisms. In most vertebrates a single insulin gene has been found, however multiple copies, some with differing gene structures, have been found in some species (e.g. rats, mice, *Xenopus laevis*, as well as teleost fishes that have undergone an ancestral whole genome duplication). In the vertebrate suborder hystricomorphs (guinea pigs and relatives), highly divergent insulin sequences are due to an accelerated rate of evolution. This is also the case in some species of New World monkeys. Several species of fish and mammals have accumulated increased amounts of sequence changes that affected sites of proteolytic processing of the prohormone precursor, resulting in either a single protein chain or two-chain proteins with an A- or B- chain that is extended to include the complete C-peptide sequence but remain biologically functional. The author concludes: “As we complete more genomes and microbiomes, it is certain that we will discover more insulin-like sequences with novel aspects to their sequences, structures, and functions. However, sequence will not tell us function. Experimental work is still needed to identify the functions of these novel insulin-like sequences, which may uncover new roles for insulin in biology”.

In a comprehensive (238 references) and well-illustrated review, Dhayalan et al. describe how the analysis of dominant diabetes-associated mutations in the human insulin gene (*INS)* provided critical insight into the folding mechanisms of proinsulin. Those mutations impair pancreatic β-cell function due to toxic misfolding of proinsulin, endoplasmic reticulum (ER) stress, intracellular proteotoxicity and impaired insulin secretion. Most mutations introduce or remove a cysteine (Cys), leading to an impaired thiol group, but non-Cys mutations affect and identify key determinants of folding efficiency. These studies suggest that the susceptibility of proinsulin to impaired foldability (“biosynthesis at the edge of non-foldability”) is one factor constraining insulin’s evolution. A similar process may occur in the natural history of Type 2 DM due to *INS* overexpression in response to insulin resistance.

In his Hypothesis and Theory article, Tatar addresses the regulation of aging in *Drosophila* and *C. elegans* by the single insulin/IGF-like receptor signaling. It is known that mutations of this receptor tyrosine kinase (InR in *Drosophila* and DAF-2 in *C. elegans)* slow aging and extend lifespan in these model invertebrates. The receptor appears to act in two modes. The first extends lifespan while slowing reproduction and reducing growth. The second strongly extends lifespan without impairing growth and reproduction; conferring “longevity assurance”. The author hypothesizes that a recent model (“threshold model” or “stability model”) for the function of receptor tyrosine kinases developed by Zinkle and Mohammadi (3) may explain how insulin receptor structure can modulate aging. Strong ligands (with fast on-rate and slow off-rate) – like DILP5 – stabilize the receptor kinase dimer and permits phosphorylation of substrates with both high and low stability thresholds of activation, resulting in robust induction of reproduction, impairing survival as a consequence of trade-offs. Weaker ligands (with slow on-rate and fast off-rate) – like DILP2 – induce only moderate kinase dimer stability and only phosphorylates sites with low stability thresholds of activation, reducing reproduction and extending lifespan by avoiding reproductive costs. Space is lacking here to go into further details of the author’s interesting speculation regarding the specific effects of the seven DILPs; this paper deserves careful reading.

Two contributions address the mode of insulin administration to diabetic patients. Masierek et al. review thoroughly the past, present and future of insulin pens, today the leading mode of insulin delivery, replacing the syringe used earlier in the last 100 years. This includes the original NovoPen in 1985, the reusable, durable pens, the disposable, prefilled pens and the modern smart insulin pens. The authors provide comprehensive tables where they review in painstaking detail all the clinical trials and all types of pens from all companies including type of insulin, participants, study design and results. This represents a considerable amount of work and may be the ultimate reference source on this topic. An original research article by Pan et al. describes a randomized, open-label, cross-over trial conducted over eight healthy adult male volunteers evaluating the absorption of needle-free insulin aspart using the QS-M needle-free jet injector from Beijing QS Medical Technology Co injected in different body parts (abdomen, upper arm and thighs). They conclude that injection sites did not affect the absorption of insulin in needle-free injections.

Two contributions addressed the pathogenesis of type 1 diabetes, a condition resulting from an absolute insulin deficiency, typically secondary to autoimmunity. In the first one, March et al. discuss the impact of nutrition and obesity on the pathogenesis of youth-onset type 1 diabetes and its complications. Over the past several decades, there has been a dramatic rise in the prevalence of overweight and obesity in pediatric populations, including among children and adolescents with type 1 diabetes (T1D). With this changing prevalence, there are youths who are now presenting with overlapping characteristics of the different types of diabetes, including features of both autoimmunity and insulin resistance, described as “double diabetes”. The authors have postulated that the increased insulin demands of obesity may accelerate the presentation of autoimmune T1D (the “accelerator hypothesis”). Various clinical studies investigating this link are described. Different pathophysiological mechanisms may explain how obesity contributes to insulitis and autoimmunity, blurring the boundary with type 2 diabetes (T2D). Insulin resistance, insulin demand and inflammation are plausible underlying mechanisms. Hormonal differences in sex steroids likely play a role in potentiating the risk for autoimmune progression. As therapeutic guidelines to manage this condition are limited, various therapeutic approaches are discussed including targeted nutritional therapies, metformin, GLP-1 agonists, DPP4-inhibitors, SGLT 1/2 inhibitors. No therapeutic options are currently approved to treat the obese youth with T1D, and future research is needed to understand which therapies might be the safest and most effective. Another review by Frommer and Kahaly examines the genetic link between T1D and autoimmune thyroid disease (AITD). T1D and AITD often cluster in individuals and families, seen as autoimmune polyendocrinopathy (AP), due to a common genetic background between TID and AITD. The major common genetic predisposition is the HLA antigens DQ2 and DQ8, tightly linked with DR3 and DR4. In addition, functional SNPs (single nucleotide polymorphisms) or rare variants of various genes that are involved in immune regulation confer susceptibility to both T1D and AITD: including *CTLA4*, *PTPN22*, *IL2Ra*, *VDR* and *TNF*. Other genes are also suspected to increase susceptibility to T1D and AITD: *CD40*, *FOXP3*, *MICA*, *INS-VNTR*, *CLEC16A*, *ERBB3*, *IFIH1*, and various cytokine genes. Furthermore, the following genes have been found in various independent GWAS studies to be associated with T1D and AITD: *BACH2*, *CCR5*, *SH2B3* and *RAC2*, indicating a strong genetic link for T1D and AITD. Consequently, all patients with T1D should be screened for AITD, and vice versa.

Two mini-reviews examine the role of two enzymes that play an important role in insulin secretion or action and metabolism, and their disorders. Possik et al. examine the role of a mammalian glycerol-3-phosphate phosphatase (recently discovered by them) in β-cell, liver and adipocyte metabolism, and cardiometabolic diseases like T2D, obesity and non-alcoholic liver steatosis. The metabolism of carbohydrates, amino acids and fats converges in generating a three-carbon molecule, glycerol, either in the free form or as glycerol-3-phosphate (Gro3P), which constitutes the backbone of glycerolipids. Free glycerol is believed to be produced and released from cells by the hydrolysis of glycerolipids in higher animals including humans. An enzyme that would generate glycerol directly from Gro3P, a Gro3P phosphatase (G3PP) was thought to be present only in plants and lower organisms. However, Prentki’s group identified in 2016 a previously described phosphoglycolate phosphatase (PGP) as the mammalian G3PP. In this review, they discuss this discovery and the most plausible physiological function of G3PP in controlling glucose, lipid and energy metabolism. They discuss the importance of G3PP in preventing glucotoxicity/nutri-stress and the control of glucose-stimulated insulin secretion (GSIS) in β-cells, in the regulation of lipogenesis in adipose tissue, and in slowing down hepatic glucose production. Finally, they address the regulation of G3PP and its role in cardiometabolic diseases. In the second mini-review, Kuefner examines the role of the secretory phospholipases A_2_ (sPLA_2_) superfamily, commonly found in mammalian tissue and snake venoms, in insulin resistance and metabolism. The sPLA_2_s are low molecular weight enzymes that hydrolyze glycerophospholipids to generate a non-esterified free fatty acid and a lysophospholipid. Twelve isoforms have been identified so far. Most studies of sPLA_2_s have addressed their roles in cardiovascular disease, inflammation, antimicrobial actions, and membrane remodeling. A discussion on how sPLA_2_s may regulate or impact glucose metabolism, insulin signaling and metabolism has been lacking. Recent research has identified 7 of the sPLA_2_s as modulators of glucose metabolism through mechanisms involved in insulin signaling and obesity. This review goes a long way in filling our gap of knowledge on these issues.

Finally, two reviews address the crosstalk between two endocrine systems, the pituitary hormones and the thyroid, and the insulin/IGF-1 systems. Schernthaner – Reiter et al. provide a brief review on the interaction of the pituitary hormone axes with glucose metabolism through direct or indirect effects on insulin secretion and function. The emphasis is on the actions on glucose metabolism of pituitary adenomas causing Cushing’s disease or acromegaly, but the authors also discuss prolactinomas, GH-deficiency, hypogonadism and hypothyroidism. Smith’s comprehensive and nicely illustrated review (134 references) discusses the two-way interplay between thyroid hormones and TSH on one hand, and growth hormone and IGF-1, on the other hand. There is a focus on Grave’s disease (GD) and thyroid-associated ophthalmopathy (TAO), and the role of IGF-1 receptors. The clinical trials of the IGF-1R blocking monoclonal antibody teprotumumab in TAO are reviewed in detail, showing an effective and well-tolerated treatment. Other potential indications are discussed.

All in all, this collection of articles represents a nice sampling of studies going from very basic to clinical applications, a suitable tribute to the 100^th^ anniversary of a pioneering hormone that remains the subject of a very fertile research field.

## Author contributions

All authors listed have made a substantial, direct, and intellectual contribution to the work and approved it for publication.
